# Incidence trend and risk factors for *campylobacter *infections in humans in Norway

**DOI:** 10.1186/1471-2458-6-179

**Published:** 2006-07-07

**Authors:** Marianne Sandberg, Karin Nygård, Hege Meldal, Paul Steinar Valle, Hilde Kruse, Eystein Skjerve

**Affiliations:** 1 The Norwegian School of Veterinary Science, P.O. Box 8146 Dep., N-0033 Oslo, Norway; 2 Norwegian Institute of Public, Health, P.O Box 4404, Nydalen N-0403 Oslo, Norway; 3 The National Veterinary Institute in Oslo, P.O. Box 8146 Dep., N-0033 Oslo, Norway

## Abstract

**Background:**

The objectives of the study were to evaluate whether the increase in incidence of campylobacteriosis observed in humans in Norway from 1995 to 2001 was statistically significant and whether different biologically plausible risk factors were associated with the incidence of campylobacteriosis in the different counties in Norway.

**Methods:**

To model the incidence of domestically acquired campylobacteriosis from 1995 to 2001, a population average random effect poisson model was applied (the trend model). To case data and assumed risk-factor/protective data such as sale of chicken, receiving treated drinking water, density of dogs and grazing animals, occupation of people in the municipalities and climatic factors from 2000 and 2001, an equivalent model accounting for geographical clustering was applied (the ecological model).

**Results:**

The increase in incidence of campylobacteriosis in humans in Norway from 1995 to 2001 was statistically significant from 1998. Treated water was a protective factor against *Campylobacter *infections in humans with an IRR of 0.78 per percentage increase in people supplied. The two-level modelling technique showed no evidence of clustering of campylobacteriosis in any particular county. Aggregation of data on municipality level makes interpretation of the results at the individual level difficult.

**Conclusion:**

The increase in incidence of *Campylobacter *infections in humans from 1995 to 2001 was statistically significant from 1998. Treated water was a protective factor against *Campylobacter *infections in humans with an IRR of 0.78 per percentage increase in people supplied. *Campylobacter *infections did not appear to be clustered in any particular county in Norway.

## Background

The reported incidence of human campylobacteriosis in Norway increased steadily, like in many other countries during the 90's and reached a maximum of 2890 cases in 2001 (64 reported cases per 100,000 inhabitants in 2001 [1,2]). The proportion of domestically acquired cases has been stable at about 40–50%. A few outbreaks have been reported, however the majority of *Campylobacter *infections seem to be sporadic as has also been observed in other developed countries [3,4].

*Campylobacter jejuni *ssp. *jejuni *(*C. jejuni*) is the most frequently isolated *Campylobacter *from humans with campylobacteriosis in Norway (personal communication, Kapperud, Georg the National Reference Laboratory, Norwegian Institute for Public Health) as observed in other developed countries [3]. However, the exact distribution of species in the reported human cases remains to be revealed and there are indications of that other species also can be important in some countries and in some age groups [5].

Case control studies and epidemiological surveys have identified risk factors for *Campylobacter *infections in humans, the most frequently revealed being untreated drinking water, consumption of broiler meat bought raw, raw milk, contact with cats and dogs and other animals and eating other types of meat at barbecue [6-12]. The risk connected to "consumption of broiler meat bought raw" might imply inadequate cooking of the meat or more likely; cross contamination in the kitchen to products that are not cooked before being eaten.

A rather high seasonal prevalence of *Campylobacter *in some surface water sources used as drinking water in Norway were found in the survey by Brennhovd et al. [13] and is also found elsewhere [14,15]. Most of the water works in Norway apply sanitation before releasing the drinking water into the net work. In some geographical areas however, there are still a high number of private water sources often localised on farms and also in houses for recreation purposes, (an aspect of the lifestyle which is common in Norway).

Case-control studies, analysis of some outbreak and comparison of strains isolated from various sources with those causing infections in humans have provided evidence that poultry represent an important risk factor [3,4,6-11,16,17]. The official import of chicken in Norway is negligible, but an unknown volume is imported through travelling and illegal import may be substantial in some areas. Because of the increased sale and consumption of fresh chicken (61% increase from 1995 to 2000), transmission of *Campylobacter *from raw or insufficient heat-treated broiler meat has been the main theory explaining the increased incidence of domestically acquired infections since 1995. However, no substantial evidence supporting this theory has been provided so far [18].

Although many studies of risk factors for campylobacteriosis in humans have been conducted world wide, there are still uncertainties related to the relative importance of the probable causal factors. As most case control studies have been matched on geography, identifying risk-factors associated with geographical location has not been possible. In regard to geographical differences in the number of sporadic and outbreak related cases, the modelling of human exposure to *Campylobacter *taking geographical areas into account can supplement traditional case-control studies and help identify risk factors of geographical origin.

Climatic variables have very often a specific geographical pattern. The association between rainfall and the incidence of *Campylobacter *infections in humans in different geographical regions in Norway has so far not been examined. Temperature is another variable that could be included in such a model in order to account for the observed north south gradient difference in the reported incidences during the summer season [7,19].

Ecological studies have featured prominently in environmental epidemiology because exposure has often been measured at the group level, or because limited resources for conducting the study prohibit collection of individual-level data [20].

The objectives of this study were to describe the reported incidence of domestically acquired campylobacteriosis from 1995 to 2001 in Norway, and to investigate whether the increase in incidence was statistically significant when accounting for variations between counties.

Moreover, we wanted to investigate the association between reported domestically acquired human *Campylobacter *infections in 2000 and 2001 and assumed biological risk factors such as consumption of chicken, proportion of people drinking treated water, density of dogs, cattle, sheep and goats, rainfall, temperature (accounting for the north south gradient in the reported incidence) and whether the population is urban/suburban or rural.

## Methods

### Case data

**Figure 1:**
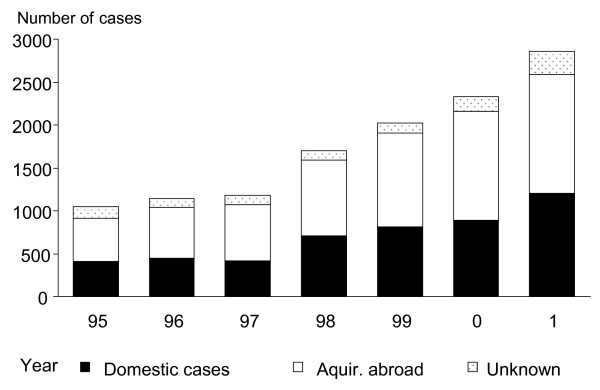
The number of reported cases of campylobacteriosis in Norway from 1995 to 2001. The histogram shows the reported number per year and by place of acquisition of infection.

Infections with *Campylobacter *are included in The Norwegian Surveillance System for Communicable Diseases (MSIS) [1] and both microbiological laboratories and all doctors in Norway are required by law to report cases to the MSIS central unit at the Norwegian Institute of Public Health. Only laboratory verified cases are reported. A case is defined as imported if the patient has a history of travelling abroad during the incubation time of *Campylobacter*, as written on the physicians notification form. We obtained information on all reported cases from 1^st ^January 1995 to 31^st ^December 2001 (Figure 1).

### Descriptive statistics

The merging of the different datasets and descriptive statistics were conducted in Excel version 2000 (Microsoft) and Stata (Stata Corp., College Station TX).

### Trend model; data and statistical analyses

In the trend analysis from 1995 to 2001, only cases reported to have acquired the infection in their home county were included in the dependent variable [1] (Table 1).

The independent variable was the year (1995–2001).

The effect of year (1995–2001) was tested using a population-averaged random-effect Poisson regression using the xtpois procedure in Stata (Stata Corp., College Station TX) with county as the random effect. The model was run with the robust estimator option (generalised estimating equation (GEE) method to adjust standard errors for clustering on county [21].

Year was regarded as statistically significant for a p-value below 0.05.

### Ecological model; data and statistical analyses

In the ecological risk factor analysis both the cases acquired within the home county as well as those domestically infected with an unknown place of infection (but home address in the respective county) were included in the dependent variable.

The independent variables were; a) The number of inhabitants in the counties ("Mid Year Number" defined by Statistics Norway) and in the municipalities in Norway from 1995 to 2001. These figures were acquired from Statistics Norway [22] (Table 1) and were used to calculate the incidence rate and to make an offset (based on the expected number of cases per inhabitant). b) Codes for seven different categories of whether the population was urban, sub urban or rural were defined using the main occupation of people in the municipality based on the "Standard for Municipality Classification" (Municipality code) from Statistics Norway [22]. The categories were (i) agricultural, (ii) agricultural and industrial, (iii) industrial, (iv) less central service and industry, (v) central service and industrial, (vi) less central service, (vii) central service municipalities. c) Kilos of broiler chicken sold per person in the different counties and municipalities in 2000 and 2001, provided by the company that has the 85% market share of the chicken broiler meat; PRIOR, Norway [23]. d) Proportion of people that receive treated drinking water in the counties and municipalities based on permanent addresses recorded in the Registry of Norwegian Waterworks per 2001 ([24]. Treatment was defined as a process that could inactivate *Campylobacter *efficiently, such as UV light, different regimes with regard to chemical treatment, and some types of filter-methods [25]. e) The number of dogs in the various counties and municipalities in 2000 and 2001 which was provided from the Norwegian Kennel Club [26]. f) The number of cattle, sheep and goat in municipalities and counties which was obtained from the Registry of Production [27] combined into one variable in the model. h) Rainfall in 2000 and 2001 in mm and temperature registrations in °C from municipalities and counties which were obtained from the Norwegian Meteorological Institute [28]. Since measuring stations for rainfall and temperature are not scattered evenly around the country, these variables were only obtained on county level. All the other included variables were collected on municipality level.

There are 19 counties and 435 municipalities in Norway.

The above mentioned variables were modelled using a population-averaged random-effect Poisson regression as described for the trend model. The selection of variables was conducted with introduction criteria of p below 0.2, and for the final model with criteria of p below 0.05, and backwards elimination.

Correlation and interaction between the variables were checked. Testing the Goodness of fit, which included checking the variance between counties, was also conducted on the final and above described random intercept model.

## Results

### Descriptive statistics

From 1995 to 2001 a total of 12327 cases of Campylobacter infections were reported in Norway, of which 4955 (40%) were acquired in Norway, 6427 (53%) were reported to be acquired abroad, and for 900 cases (7%) for which information on place of infection was not available.

Of the 4955 domestically acquired cases, 56% were male and 44% female (0.2% were unknown).

The median age among the cases was 29 years, but 16.9% of the cases were in the age group 0 to 4 years old, where boys accounted for 61.4% of the cases. Males dominated in all age groups except among cases over 70 years all, however among those 20–29 years old the difference was small (51.9% males and 48.1% females). The median age increased from 25 to 32 in the period; 1995 to 2001.

Sixty-six percent of the reported cases from 1995 to 2001 were reported during June, July and August regardless of age or sex.

### The trend model

**Figure 2:**
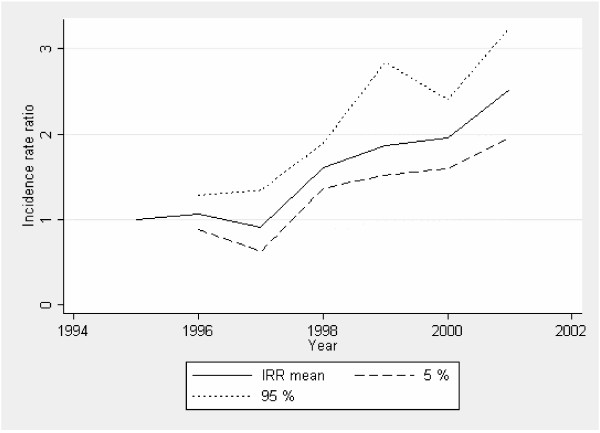
Time trend of *Campylobacter *incidence in Norway; Results from population-averaged random-effect Poisson regression, showing results as incidence rate ratios (IRR) with 95% confidence interval (CI).

From 1995 to 2001, a total of 4955 domestically acquired cases of human campylobacteriosis were reported. The increase in the annually reported number of cases acquired domestically and within the home county was statistically significant from 1998 and thereafter (Figure 2).

### The ecological model

In 2000 and 2001, the total number of domestically acquired campylobacteriosis cases reported was 2109, which corresponds to an annual incidence of 23.4 per 100,000 inhabitants.

The mean quantity of chicken sold from Prior in 2000 and 2001 was 7.69 kg per inhabitant (95% CI; 3.72–11.48) (Table 2). According to the Norwegian Institute of Public Health categorisation, about 90% of the inhabitants received treated drinking water from water works in 2001 (Table 2). The density of dogs and grazing animals in the 20 counties, as well as rainfall (median) and temperature (median) is shown in Table 2.

The final poisson regression model (Table 3) included treated drinking water as a protective factor with an incidence rate ratio (IRR) of 0.776 percent increase in people with treated water (95% Confidence Interval; 0.630, 0.954).

Rainfall was a risk factor for *Campylobacter *infection with an IRR of 1.006 per mm (1.005, 1.007).

No high degree of correlation was found amongst the included variables. The variation at the county level was low.

## Discussion

No differences in the culturing of human stool samples for *Campylobacter *were implemented in the period 1995–2001, the results from the trend model are therefore assumed to fairly represent the real trend (Figure 2).

Similar trends for age and sex as in this dataset were reported in the period 1979 to 1988 [7]. Numbers can, however, not be directly compared between these periods, as changes in the reporting system occurred when the infection became notifiable in 1995. The high number of recorded incidences in some municipalities at the west coast of Norway was partly due to outbreaks during summertime [1]. The number of reported cases is regarded as a highly biased estimate of the true incidence of campylobacteriosis due to a probable major underreporting of cases. Classification of cases in the county where the infection was acquired is biased to some extent in both datasets as a consequence of ignorance about the source of infection.

Treated drinking water came out as a protective factor, however categorisation of the number of people that received disinfected or not disinfected water in the dataset was complicated; the main problem being that some waterworks supplied water from different sources. All the groundwater sources were expected to supply water of high quality [25,29]. Therefore all the people that received water from groundwater and from the waterworks that had sufficient disinfections of the water to kill *Campylobacter *were counted as having received treated drinking water. Table 2 shows that in some counties (South Middle, South West and in the Northern part of Norway) only 50% of the inhabitants receive treated drinking water.

Drinking water from waterworks is not always representative of the drinking-water quality received by the consumer. Besides being exposed to drinking water in their home, people could be exposed to untreated water during vacations and week-end trips into forests, mountains and to recreational houses with private water supplies.

A further problem is the network of water pipes being very old in some areas and therefore of poor quality. Events such as pressure drops in the water pipe system can lead to influx of contaminants from the surroundings outside the water pipes. A Norwegian cohort study, where such pressure drops were recorded indicated that there was an association between low-pressure incidents and diarrhoea in humans [30]. Influx of contaminated ground water into the water pipelines due to bad pipe quality and/or low pressure events has also been shown to be a serious problem elsewhere [31,32].

Unlike the other independent variables, rainfall and temperature were aggregated at a county level which introduces more uncertainty and difficulties in the interpretation of the results. Currently the registration spots for rainfalls are distributed unequally in the different municipalities. As far as the authors are aware, the number of measuring spots is not connected to the size of the municipalities and consequently the resolution of the measurements could be very different from one geographical area to another. An ecological or aggregate study focuses on the comparison of groups rather than individuals. The fact that we do not know the joint distribution of the study factors and the disease incidence within each group, means that an ecological study could lead us into the classic ecological fallacy (i.e. making causal interferences about an individual phenomenon or process when the observations are made at group level (aggregated) [20]. Rainfall is, however, a very plausible explanation for an increased *Campylobacter *incidence in humans, resulting from the washing-out of *Campylobacter *contaminated faeces from the soil into the drinking water sources for humans and animals. Even though the result from the multivariable ecological model was not statistically significant for geographical location (counties) (Table 3), there was an association between higher rainfall and the incidence of *Campylobacter *infections in humans. Generally, more rain falls on the Southwest coast of Norway than in the rest of the country and that was also the case during the study period (Table 2).

A major problem with regard to intervention and control of campylobacteriosis in humans is the high number of potential sources of infection. Since broiler chicken has been regarded as a very important source of infection and also as a possible point of control some countries have implemented Action Plans or similar activities in broiler production [2]. A great step forward with regard to the lowering of the *Campylobacter *prevalence in Norwegian broiler flocks has taken place from 2001 up to the present time [33,34]. The number of *Campylobacter *counts on the carcasses that enters the market has also been reduced by implementing the freezing of the infected carcasses for 3 weeks as a risk-reducing measure in the Norwegian Action Plan against *Campylobacter *[35].

In addition to food there are also several environmental *Campylobacter *sources including water, for drinking and recreation, and contact with food production animals and pets, and exposure to soil. How significant the different risk factors for *Campylobacter *infections are in different countries is hard to evaluate. Surface drinking water supplies seems to be a risk factor for high *Campylobacter *incidence. Disinfection of drinking water at the water works is, as mentioned earlier not always sufficient, and improvement of the water pipelines or the control of pressure drops are interventions that should be implemented. Modern equipment for control of pressure drops is available on the market [31].

Most of the *Campylobacter *infections are still regarded as not being a part of an outbreak but sporadic. The relative importance of the different risk factors for sporadic *Campylobacter *infections is likely to be variable and the importance of them individually will vary over time and space. Effort should therefore be taken to control all the known risk factors for *Campylobacter *infections in man. There are however, many sources of *Campylobacter *bacteria from which it seems impossible to control all possible transmission routes i.e. contact with pets and exposure to contaminated soil. Therefore, in order to prevent *Campylobacter *infections, we conclude that it is very important to maintain a satisfactory overall level of hygiene, especially for children and people with compromised immunological status.

## Conclusion

The increase in incidence of campylobacteriosis in humans from 1995 to 2001 was statistically significant from 1998 and thereafter.

Treated water was a protective factor against *Campylobacter *infections in humans with an IRR of 0.78 per percentage increase in people supplied.

The two-level modelling technique revealed that campylobacteriosis was not clustered in any particular county which for practical purposes indicates that there are not any specific local risk factors for human *Campylobacter *infections in Norway. This is consistent with the hypothesis that several risk factors are of differing importance at different time for the incidence of sporadic *Campylobacter *infections.

## Competing interests

The author(s) declare that they have no competing interests.

## Authors' contributions

All of the listed authors contributed to the manuscript, in the planning and design of the study, in collecting data and in literature search. HM contributed to a great extent in collecting data and with the descriptive statistics. KN, PSV and ES participated in the analytical part of the study. First author had the main responsibility for; the analytical part and writing of the manuscript.

## Pre-publication history

The pre-publication history for this paper can be accessed here:



## Figures and Tables

**Table 1:** The reported incidence (cases/100 000) of human campylobacteriosis acquired domestically (within home county) from 1995 to 2001.

County	Human cases						
	1995	1996	1997	1998	1999	2000	2001

1	3.8	1.2	4.9	6.5	10.5	11.6	13.9
2	1.8	3.6	3.6	5.0	8.6	6.4	13.5
3	4.7	6.9	8.0	10.8	12.1	13.2	18.0
4	5.9	8.1	3.8	10.2	11.8	12.8	11.7
5	9.3	9.9	6.0	11.5	18.6	18.6	18.0
6	5.7	5.7	2.6	11.1	10.6	15.6	10.5
7	8.8	4.9	8.2	13.8	13.7	15.4	12.6
8	2.5	4.3	5.5	14.0	12.1	17.5	10.3
9	3.0	8.0	1.0	14.8	9.8	9.8	25.3
10	4.7	10.6	20.4	8.5	14.9	31.4	35.7
11	17.1	14.2	12.1	22.6	24.5	25.6	32.5
12	11.6	9.6	3.3	17.2	21.2	20.6	26.5
14	10.2	11.1	7.4	10.2	26.0	17.7	34.4
15	7.5	11.6	7.4	8.7	9.9	11.1	13.9
16	12.5	12.0	14.7	20.4	16.4	14.8	20.8
17	12.6	10.2	3.8	11.8	10.2	18.9	18.9
18	12.8	16.2	9.2	11.3	15.1	21.8	22.2
19	17.9	19.8	19.9	22.6	10.6	14.6	11.2
20	6.5	7.9	8.0	5.3	1.4	9.5	0

**Table 2:** Number of domestic human campylobacteriosis cases, amount of fresh and frozen broiler meat sold (kg/person), proportion of  people (median) that received untreated drinking water, the number of dogs and grazing animals, the rainfall in mm (median) and temperature in Celsius (median) by county for 2000 and 2001.

County	Cases	Incidence rate	Chicken (kg/person) sold	Proportion (median) of people with treated water	No. of grazing animals	No. of dogs (2001)	Rain in mm (median)	Temperature °C
1	87	0.00035	6.579	0.785	24095	12154	84.300	7.610
2	180	0.00038	8.929	0.945	28916	21830	108.390	7.610
3	232	0.00046	11.742	1	117	13063	82.930	8.000
4	51	0.00027	9.216	0.740	115474	13565	62.240	7.580
5	81	0.00044	9.249	0.510	206375	12293	57.200	6.800
6	84	0.00035	9.449	0.800	82591	12024	85.920	7.600
7	74	0.00035	11.463	0.940	14083	8817	85.920	7.600
8	70	0.00042	11.182	0.705	30187	8270	94.990	7.360
9	52	0.00051	10.935	0.520	43243	5105	142.770	7.760
10	133	0.00085	8.694	0.750	51255	6079	171.760	5.340
11	273	0.00073	6.828	0.900	325486	11664	185.570	5.340
12	256	0.00059	8.012	0.640	175359	12389	174.470	7.440
14	78	0.00072	7.551	0.600	199028	3226	162.520	6.250
15	84	0.00034	7.445	0.860	159174	5779	113.000	4.170
16	145	0.00070	5.357	0.760	140270	10942	91.760	6.760
17	50	0.00039	3.634	0.760	133651	7586	82.710	6.760
18	126	0.00053	4.651	0.820	157482	9706	97.540	5.780
19	46	0.00030	4.488	0.570	112381	5661	86.570	6.430
20	7	0.00009	5.641	0.950	20357	3979	36.820	8.340

**Table 3:** Results from the final ecological model, given as Incidence Rate Ratios (IRR).

Variables	IRR	P	95% CI (IRR)
Proportion (median) of people with treated water	0.776	0.016	0.630, 0.954
Rain in mm (median)	1.006	0.000	1.005, 1.007
Constant	0.712	0.006	0.559, 0.906
